# Supplementary material for: Involvement of superior colliculus in complex figure detection of mice
Source: eLife. 2024 Jan 25;13:e83708. doi: 10.7554/eLife.83708 (PMC10810606; doi:10.7554/eLife.83708)
Supplement: Figure 1—source data 1. [file elife-83708-fig1-data1.docx]

**Figure 1—source data 1. Statistics**

| **Panel** | **Comparison** | **Mean & SEM** | **Test** | **Statistic** | **p-value** | **Correction** |
| --- | --- | --- | --- | --- | --- | --- |
| E | Effect of optogenetics on contrast task (N = 5 mice) | 17 ms: 75 ± 3%  33 ms: 76 ± 4%  50 ms: 74 ± 4%  66 ms: 77 ± 6%  100 ms: 82 ± 4%  133 ms: 91 ± 3%  No opto: 89 ± 3% | One-way repeated measures ANOVA,  Post-hoc Tukey-Kramer | Main: F(6) = 5.16 | Main: 0.005 **  17 vs. no: 0.027 *  33 vs. no: 0.062  50 vs. no: 0.022 *  66 vs. no: 0.095  100 vs. no: 0.551  133 vs. no: 0.999 | Main: Bonferroni for 3 tasks |
|  | Inflection point - Contrast | 99 ± 8 ms (SD) | Bootstrap (n = 1000) |  |  |  |
|  | Effect of optogenetics on orientation task (N = 6 mice) | 33 ms: 61 ± 2%  66 ms: 62 ± 3%  100 ms: 60 ± 3%  133 ms: 65 ± 4%  166 ms: 67 ± 3%  200 ms: 70 ± 3%  No opto: 73 ± 2% | One-way repeated measures ANOVA,  Post-hoc Tukey-Kramer | Main: F(6) = 3.55 | Main: 0.027 *  33 vs. no: 0.034 *  66 vs. no: 0.051  100 vs. no: 0.021 *  133 vs. no: 0.322  166 vs. no: 0.566  200 vs. no: 0.975 | Main: Bonferroni for 3 tasks |
|  | Inflection point - Orientation | 156 ± 35 ms (SD) | Bootstrap |  |  |  |
|  | Effect of optogenetics on phase task (N= 6 mice) | 33 ms: 60 ± 4%  66 ms: 52 ± 4%  100 ms: 64 ± 2%  133 ms: 63 ± 6%  166 ms: 69 ± 3%  200 ms: 71 ± 3%  No opto: 73 ± 2% | One-way repeated measures ANOVA, Post-hoc Tukey-Kramer | Main: F(6) = 5.03 | Main: 0.003**  33 vs. no: 0.115  66 vs. no: 0.001 **  100 vs. no: 0.435  133 vs. no: 0.702  166 vs. no: 0.976  200 vs. no: 0.999 | Main: Bonferroni for 3 tasks |
|  | Inflection point – Phase | 134 ± 30 ms (SD) | Bootstrap |  |  |  |

**Figure 1—figure supplement 2**

| **Panel** | **Comparison** | **Mean & SEM** | **Test** | **Test Statistic** | **p-value** | **Correction** |
| --- | --- | --- | --- | --- | --- | --- |
| G,inset | Firing rate for laser on vs. laser off  (170 units from  3 GAD2Cre mice) | Off: 13.6 ± 1.0 sp/s  On: 3.3 ± 0.7 sp/s | LME: *rate ~ opto + (1\|unit) + (opto\|session)* | F(1, 338) = 55.58 | < 0.001 *** | none |

**Figure 1—figure supplement 3**

| **Panel** | **Comparison** | **Mean & SEM** | **Test** | **Test Statistic** | **p-value** | **Correction** |
| --- | --- | --- | --- | --- | --- | --- |
| E | Evoked firing rate for laser on vs. laser off  (109 units from  4 WT mice) | Off: 10.0 ± 0.9 sp/s  On: 9.6 ± 0.9 sp/s | LME: *rate ~ opto + (1\|unit) + (opto\|session)* | F(1, 402) = 16.83 | < 0.001 *** | none |

**Figure 1—figure supplement 4**

| **Panel** | **Comparison** | **Mean & SEM** | **Test** | **Statistic** | **p-value** | **Correction** |
| --- | --- | --- | --- | --- | --- | --- |
| B | Contrast - Hits | 17 ms: 58 ± 5 %  33 ms: 59 ± 6 %  50 ms: 58 ± 4 %  67 ms: 60 ± 5 %  100 ms: 67 ± 4 %  133 ms: 75 ± 3 %  No opto: 78 ± 4 % | One-way repeated measures ANOVA, Post-hoc Dunnett | Main effect:  F = 9.10 | Main: 0.00011 ***  17 vs. no: 0.00018 ***  33 vs. no: 0.00024 ***  50 vs. no: 0.00013 ***  67 vs. no: 0.00057 ***  100 vs. no: 0.045 *  133 vs. no: 0.88 | Main: Bonferroni for 3 tasks |
|  | Contrast - Errors | 17 ms: 20 ± 3 %  33 ms: 18 ± 3%  50 ms: 20 ± 4 %  67 ms: 19 ± 5 %  100 ms: 15 ± 3 %  133 ms: 8 ± 4 %  No opto: 10 ± 3 % | One-way repeated measures ANOVA, Post-hoc Dunnett | Main effect:  F = 4.23 | Main: 0.016 *  17 vs. no: 0.022 *  33 vs. no: 0.063  50 vs. no: 0.018 *  67 vs. no: 0.055  100 vs. no: 0.33  133 vs. no: 0.99 | Main: Bonferroni for 3 tasks |
|  | Contrast - Misses | 17 ms: 22 ± 6 %  33 ms: 23 ± 6 %  50 ms: 22 ± 3 %  67 ms: 21 ± 5 %  100 ms: 17 ± 4 %  133 ms: 17 ± 6 %  No opto: 12 ± 3 % | One-way repeated measures ANOVA, Post-hoc Dunnett | Main effect:  F = 2.51 | Main: 0.15  17 vs. no: 0.045 *  33 vs. no: 0.021 *  50 vs. no: 0.040 *  67 vs. no: 0.060  100 vs. no: 0.48  133 vs. no: 0.51 | Main: Bonferroni for 3 tasks |
|  | Orientation - Hits | 17 ms: 37 ± 7 %  33 ms: 36 ± 7 %  50 ms: 39 ± 6 %  67 ms: 38 ± 8 %  100 ms: 43 ± 7 %  133 ms: 45 ± 8 %  No opto: 51 ± 2 % | One-way repeated measures ANOVA, Post-hoc Dunnett | Main effect:  F = 2.98 | Main: 0.055  17 vs. no: 0.014 *  33 vs. no: 0.011 *  50 vs. no: 0.040 *  67 vs. no: 0.025 *  100 vs. no: 0.28  133 vs. no: 0.62 | Main: Bonferroni for 3 tasks |
|  | Orientation - Errors | 17 ms: 23 ± 5 %  33 ms: 22 ± 3 %  50 ms: 26 ± 2 %  67 ms: 22 ± 4 %  100 ms: 21 ± 2 %  133 ms: 20 ± 3 %  No opto: 19 ± 2 % | One-way repeated measures ANOVA, Post-hoc Dunnett | Main effect:  F = 1.33 | Main: 0.81  17 vs. no: 0.45  33 vs. no: 0.74  50 vs. no: 0.080  67 vs. no: 0.84  100 vs. no: 0.90  133 vs. no: 1.00 | Main: Bonferroni for 3 tasks |
|  | Orientation - Misses | 17 ms: 40 ± 12 %  33 ms: 42 ± 9 %  50 ms: 35 ± 7 %  67 ms: 41 ± 10 %  100 ms: 36 ± 8 %  133 ms: 35 ± 10 %  No opto: 31 ± 4 % | One-way repeated measures ANOVA, Post-hoc Dunnett | Main effect:  F = 1.00 | Main: 1.00  17 vs. no: 0.37  33 vs. no: 0.22  50 vs. no: 0.91  67 vs. no: 0.29  100 vs. no: 0.83  133 vs. no: 0.90 | Main: Bonferroni for 3 tasks |
|  | Phase - Hits | 17 ms: 32 ± 6 %  33 ms: 28 ± 5 %  50 ms: 38 ± 7 %  67 ms: 37 ± 6 %  100 ms: 45 ± 8 %  133 ms: 49 ± 7 %  No opto: 49 ± 3 % | One-way repeated measures ANOVA, Post-hoc Dunnett | Main effect:  F = 6.1 | Main: 0.00050  17 vs. no: 0.0037 **  33 vs. no: 0.00050 ***  50 vs. no: 0.12  67 vs. no: 0.081  100 vs. no: 0.90  133 vs. no: 1.00 | Main: Bonferroni for 3 tasks |
|  | Phase - Errors | 17 ms: 23 ± 5 %  33 ms: 28 ± 6 %  50 ms: 24 ± 3 %  67 ms: 23 ± 6 %  100 ms: 21 ± 2 %  133 ms: 20 ± 4 %  No opto: 18 ± 2 % | One-way repeated measures ANOVA, Post-hoc Dunnett | Main effect:  F = 1.7 | Main: 0.47  17 vs. no: 0.67  33 vs. no: 0.029  50 vs. no: 0.47  67 vs. no: 0.56  100 vs. no: 0.89  133 vs. no: 0.98 | Main: Bonferroni for 3 tasks |
|  | Phase - Misses | 17 ms: 46 ± 10 %  33 ms: 43 ± 10 %  50 ms: 38 ± 9 %  67 ms: 40 ± 9 %  100 ms: 34 ± 10 %  133 ms: 31 ± 10 %  No opto: 33 ± 4 % | One-way repeated measures ANOVA, Post-hoc Dunnett | Main effect:  F = 2.2 | Main: 0.20  17 vs. no: 0.091  33 vs. no: 0.24  50 vs. no: 0.82  67 vs. no: 0.65  100 vs. no: 1.00  133 vs. no: 1.00 | Main: Bonferroni for 3 tasks |

**Figure 1—figure supplement 5**

| **Panel** | **Comparison** | **Mean & SEM** | **Test** | **Statistic** | **p-value** | **Correction** |
| --- | --- | --- | --- | --- | --- | --- |
| C | Effect of optogenetics on maximum lick rate (N = 8 mice) | 17 ms: 7.4 ± 0.4 l/s  33 ms: 7.5 ± 0.6 l/s  50 ms: 7.9 ± 0.5 l/s  67 ms: 7.3 ± 0.5 l/s  100 ms: 7.2 ± 0.4 l/s  133 ms: 7.3 ± 0.6 l/s  166 ms: 7.2 ± 0.6 l/s  200 ms: 7.5 ± 0.6 l/s  No opto: 6.9 ± 0.4 l/s | One-way repeated measures ANOVA, Post-hoc Tukey-Kramer | Main effect:  F(8) = 1.37 | Main: 0.235  17 vs. no: 0.552  33 vs. no: 0.852  50 vs. no: 0.091  66 vs. no: 0.946  100 vs. no: 0.988  133 vs. no: 0.940  166 vs no: 0.999  200 vs no: 0.799 | None |
| D | Effect of optogenetics on reaction times in contrast task (N = 5 mice) | 17 ms: 756 ± 100 ms  33 ms: 745 ± 82 ms  50 ms: 749 ± 87 ms  66 ms: 684 ± 67 ms  100 ms: 705 ± 54 ms  133 ms: 695 ± 44 ms  No opto: 632 ± 27 ms | One-way repeated measures ANOVA, Post-hoc Tukey-Kramer | Main effect:  F(6) = 1.74 | Main: 0.469  17 vs. no: 0.180  33 vs. no: 0.246  50 vs. no: 0.225  66 vs. no: 0.926  100 vs. no: 0.730  133 vs. no: 0.905 | Main: Bonferroni for 3 tasks |
|  | Effect of optogenetics on reaction times in orientation task (N = 6 mice) | 33 ms: 800 ± 70 ms  66 ms: 763 ± 51 ms  100 ms: 789 ± 46 ms  133 ms: 798 ± 74 ms  166 ms: 773 ± 63 ms  200 ms: 771 ± 58 ms  No opto: 710 ± 57 ms | One-way repeated measures ANOVA, Post-hoc Tukey-Kramer | Main effect:  F(6) = 1.75 | Main: 0.437 33 vs. no: 0.118  66 vs. no: 0.666  100 vs. No: 0,190  133 vs. no: 0.131  166 vs. no: 0.340  200 vs. no: 0.365 | Main: Bonferroni for 3 tasks |
|  | Effect of optogenetics on reaction times in phase task (N = 6 mice) | 33 ms: 808 ± 84 ms  66 ms: 823 ± 59 ms  100 ms: 831 ± 69 ms  133 ms: 817 ± 61 ms  166 ms: 801 ± 43 ms  200 ms: 808 ± 52 ms  No opto: 691 ± 55 ms | One-way repeated measures ANOVA, Post-hoc Tukey-Kramer | Main effect:  F(6) = 3.06 | Main: 0.056 33 vs. no: 0.068  66 vs. no: 0.028*  100 vs. no: 0.016*  133 vs. no: 0.040*  166 vs. no: 0.103  200 vs. no: 0.069 | Main: Bonferroni for 3 tasks |
