# Supplementary material for: Involvement of superior colliculus in complex figure detection of mice
Source: eLife. 2024 Jan 25;13:e83708. doi: 10.7554/eLife.83708 (PMC10810606; doi:10.7554/eLife.83708)
Supplement: Figure 2—source data 1. [file elife-83708-fig2-data1.docx]

**Figure 2—source data 1. Statistics**

| **Panel** | **Comparison** | **Mean & SEM** | **Test** | **Statistic** | **p-value** | **Correction** |
| --- | --- | --- | --- | --- | --- | --- |
| I | Figure vs. Ground – Contrast  (22 units from 3 mice) | n.a. (too many comparisons) | Permutation test of clustered LME: *Normalized rate ~ stimulus + (1\|mouse) + (1\|session) + (1\|unit)* | F(1, 61) | <0.05 from 70-99 ms | Clustered LME-statistic, see Methods section |
|  | Figure vs. Ground – Orientation  (49 units from 5 mice) | n.a. (too many comparisons) | Permuation test of clustered LME: *Normalized rate ~ stimulus + (1\|mouse) + (1\|session) + (1\|unit)* | F(1, 96) | <0.05 from 83-130 ms | Clustered LME-statistic, see Methods section |
|  | Figure vs. Ground – Phase  (32 units from 5 mice) | n.a. (too many comparisons) | Permutation test of Clustered LME: *Normalized rate ~ stimulus + (1\|mouse) + (1\|session) + (1\|unit)* | F(1, 62) | No significant clusters | Clustered LME-statistic, see Methods section |
| J | Onset modulation - Contrast | 67 ms | Curve fitting |  |  |  |
|  | Onset modulation - Orientation | 75 ms | Curve fitting |  |  |  |

**Figure 2—figure supplement 1**

| **Panel** | **Comparison** | **Mean & SEM** | **Test** | **Statistic** | **p-value** | **Correction** |
| --- | --- | --- | --- | --- | --- | --- |
| D | d-prime for  - Hit vs. Error  - Orientation vs. Phase  (5/5 and 8/8 units for orientation hit/error and phase hit/error, respectively) | Orientation Hit:  0.671 ± 0.429  Orientation Error:  -0.353 ± 0.496  Phase Hit:  0.607 ± 0.439  Phase Error:  -0.398 ± 0.491 | LME:  *dprime ~ 1 + Response + Task* | **Main:**  Resp.: F(1,19) = 4.645  Task: F(1,19) = 0.014  **Post Hoc:**  Resp. (Orientation)  T(7) = 1.569  Resp. (Phase)  T(111) = 1.480 | **Main :**  0.044*  0.906  **Post-Hoc :**  0.161  0.167 | None |
| E | Variance of depth of visual vs. MS cells  Difference in depth of visual vs. MS cells  (99 and 8 units for visual and MS cells, respectively) | Visual: 338 ± 190 um (SD)  Multisensory: 416 ± 92 um (SD) | Two-sample  F-test, two-tailed  Mann-Whitney  U-test, two-tailed | F(7, 98) = 0.231  Z = 1.352 | 0.046  0.177 | None  None |
| F | Correlation of eye speed with neural activity vs. correlation coefficient of zero | Coefficients:  -0.025 ± 0.031 | One-sample t-test, two-tailed | T(3) = -0.789 | 0.488 | None |
