# Supplementary material for: Involvement of superior colliculus in complex figure detection of mice
Source: eLife. 2024 Jan 25;13:e83708. doi: 10.7554/eLife.83708 (PMC10810606; doi:10.7554/eLife.83708)
Supplement: Figure 3—source data 1. [file elife-83708-fig3-data1.docx]

**Figure 3—source data 1. Statistics**

| **Panel** | **Comparison** | **Mean & SEM** | **Test** | **Statistic** | **p-value** | **Correction** |
| --- | --- | --- | --- | --- | --- | --- |
| B | Figure vs. Ground – Orientation  (12 units from 3 mice) | n.a. (too many comparisons) | Clustered LME:  *Normalized rate ~ stimulus + (1\|mouse) + (1\|session) + (1\|unit)* | F(1, 22) | <0.05 from 80-103 ms | Clustered LME-statistic |
|  | Figure vs. Ground – Phase  (14 units from 3 mice) | n.a. (too many comparisons) | Clustered LME:  *Normalized rate ~ stimulus + (1\|mouse) + (1\|session) + (1\|unit)* | F(1, 26) | No significant clusters | Clustered LME-statistic |
| D | Orientation decoder | n.a. (too many comparisons) | Binomial test, two-tailed | n.a.  (N = 2000) | 0-50 ms: 1  10-60 ms: 0.468  20-70 ms: 1  30-80 ms: 1  40-190 ms: <0.001 ***  150-200 ms: 0.024 *  160-210 ms: 1  170-220 ms: 1  180-230 ms: 1  190-240 ms: 0.025 *  200-250 ms: 0.230  210-260 ms: 0.002 **  220-270 ms: 0.771  230-280 ms: 1 | Bonferroni-Holm |
|  | Phase decoder | n.a. (too many comparisons) | Binomial test, two-tailed | n.a.  (N = 2000) | 0-50 ms: 0.345  10-60 ms: 0.118  20-70 ms: < 0.001 ***  30-80 ms: 0.336  40-90 ms: < 0.001 ***  50-100 ms: < 0.001 ***  60-110 ms: 0.126  70-120 ms: 0.345  80-130 ms: 0.257  90-140 ms: 0.336  100-150 ms and later bins: < 0.001 *** | Bonferroni-Holm |
| E | Relative model weight of neurons with RF inside vs. RF edge, for each task | Ori / RF inside:  0.214 ± 0.035  Ori / RF edge:  0.220 ± 0.064  Phase / RF inside:  0.049 ± 0.059  Phase / RF edge:  0.149 ± 0.069 | LME:  *Modelweight ~ Task + RF + (1\|Unit) + (1\|Session) + (1\|Mouse)* | **Main:**  Task: F(1, 104) = 5.80  RF: F(1, 104) = 0.27  **Post Hoc:**  RF (Orientation)  F(1, 59) = 0.01  RF (Phase)  T(1, 44) = 0.97 | 0.018 *  0.608  0.941  0.329 | None |
| F | Orientation mean d’ | Mean d’ = 0.302 | Bootstrap (n = 5000), two-tailed | CI 99.9%  -0.147 – 0.137 | < 0.001 *** | None |
|  | Phase mean d’ | Mean d’ = 0.090 | Bootstrap (n = 5000), two-tailed | CI 95%  -0.085 – 0.085 | < 0.05 * | None |
