# Supplementary material for: Involvement of superior colliculus in complex figure detection of mice
Source: eLife. 2024 Jan 25;13:e83708. doi: 10.7554/eLife.83708 (PMC10810606; doi:10.7554/eLife.83708)
Supplement: Figure 4—source data 1. [file elife-83708-fig4-data1.docx]

**Figure 4—source data 1. Statistics**

| **Panel** | **Comparison** | **Mean & SEM** | **Test** | **Statistic** | **p-value** | **Correction** |
| --- | --- | --- | --- | --- | --- | --- |
| C | d-prime for  - Hit vs. Error  - Orientation vs. Phase  (61 and 46 units for orientation and phase respectively) | Orientation Hit:  0.404 ± 0.078  Orientation Error:  -0.093 ± 0.138  Phase Hit:  0.325 ± 0.098  Phase Error:  0.158 ± 0.131 | LME:  *dprime ~ 1 + Response + Task + (1\|Unit) + (1\|Session) + (1\|Mouse)* | **Main:**  Resp.: F(1,191) = 10.43  Task: F(1,191) = 0.52  **Post Hoc:**  Resp. (Orientation)  F(1, 101) = 11.09  Resp. (Phase)  F(1, 89) = 1.07 | **Main:**  0.001 **  0.473  **Post-Hoc :**  0.001 **  0.303 | None |
